# Monthly trends, determinants, and forecasting of perinatal mortality in Ghana: a comparison of ARIMA, BPNN, DLNN, and GRNN models

**DOI:** 10.3389/frph.2026.1849802

**Published:** 2026-07-10

**Authors:** Agyei Helena Lartey, Denis Dekugmen Yar, Ama Asamaniwa Attua, Godfred Nyanney, Akuffo Samuel Tete Manukure, Isaac Takyi Boahen, Theophilus Oduro Kankam, Collins Mawuli Bakudie

**Affiliations:** 1 Department of Public Health Education, University of Skills Training and Entrepreneurial Development, Mampong, Ghana; 2 Department of Biological Sciences, University of Skills Training and Entrepreneurial Development, Mampong, Ghana; 3 Department of Health Information, Ghana Health Service, Mampong, Ghana

**Keywords:** perinatal mortality, antenatal care coverage, ARIMA, forecasting, neural networks, hypertensive disorders in pregnancy, maternal health, ghana

## Abstract

**Background:**

Perinatal mortality is a critical indicator of the quality of maternal and newborn care across sub-Saharan Africa. A recent systematic review and meta-analysis estimated Ghana's pooled perinatal mortality rate at 44.8 per 1,000 births, highlighting ongoing barriers to meeting Sustainable Development Goal targets for neonatal survival.

**Methods:**

We conducted a retrospective, hospital-based time series analysis of 192 monthly observations from January 2010 through December 2025. Perinatal mortality rate (PMR) was defined as the sum of stillbirths and early neonatal deaths per 1,000 births. Stationarity was evaluated using the Augmented Dickey Fuller (ADF) test. Forecasting performance was compared across four models—ARIMA, backpropagation neural network (BPNN), deep learning neural network (DLNN), and generalized regression neural network (GRNN)—with model validation performed on a 2025 temporal holdout.

**Results:**

The hospital recorded 46,108 live births and 1,152 perinatal deaths, giving an overall PMR of 24.98 per 1,000 births. The undifferenced monthly PMR series was borderline non-stationary (ADF statistic −2.695; *p* = 0.075), while the first-differenced series was stationary (ADF statistic −7.118; *p* < 0.001). The best-performing ARIMA model on the 2025 holdout was ARIMA (3, 0, 0). Test-set RMSE values were 11.74 for ARIMA, 14.98 for BPNN, 12.87 for DLNN, and 13.33 for GRNN. In ecological monthly models, higher ANC coverage was associated with lower PMR, whereas higher hypertension burden was associated with higher PMR.

**Conclusion:**

Perinatal mortality declined over the long term but remained unstable. Among the evaluated models, ARIMA showed the best out-of-sample accuracy, while GRNN was the strongest neural-network comparator. Forecasts should be interpreted as operational projections rather than causal predictions.

What is already known on this topic?

Perinatal mortality remains high in many low- and middle-income countries, and stillbirths plus early neonatal deaths continue to contribute substantially to under-5 mortality in Ghana ( 1– 6).

What this study adds

This study provides a 16-year monthly hospital time series, compares classical time-series forecasting with three neural-network approaches, and demonstrates that better ANC coverage and lower hypertension burden track with lower monthly PMR in this setting.

How this study might affect research, practice, or policy

Monthly PMR surveillance may help maternity hospitals monitor service quality, and ARIMA-based operational forecasting may assist planning for high-risk periods while service-improvement efforts focus on ANC utilization and maternal complication control.

## Introduction

Perinatal mortality, defined as stillbirths and early neonatal deaths, remains a critical public health outcome because it captures the combined effect of maternal health, intrapartum care, and newborn services (1–3, 5). In low- and middle-income countries, perinatal deaths continue to occur at disproportionately high rates, and progress has been slower than for post-neonatal child survival (1, 2).

In Ghana, neonatal mortality remains an important contributor to under-5 mortality. The 2022 Ghana Demographic and Health Survey reported a neonatal mortality rate of 17 deaths per 1,000 live births for the five years preceding the survey (7). Ghana-specific reviews also show that perinatal mortality varies substantially by setting, referral profile, and quality of obstetric and neonatal care (6, 8).

Several determinants of perinatal mortality have been described in Ghana and across sub-Saharan Africa, including inadequate antenatal care, hypertensive disorders of pregnancy, prematurity, low birth weight, delayed care-seeking, and constrained referral systems (8–11). Monthly hospital data can therefore be informative for both epidemiological monitoring and operational planning.

Time-series methods are increasingly used to evaluate maternal and newborn health indicators. ARIMA models remain attractive for comparatively short but regular health series because they are transparent and interpretable (12–14). Neural-network approaches such as BPNN, DLNN, and GRNN can model nonlinear dynamics and have been used in epidemiological forecasting, although their performance depends strongly on data volume, feature design, and forecast horizon (15–17).

This study analyzed monthly hospital statistics from Mampong Government Maternity Hospital for 2010–2025 to estimate the prevalence and long-term trend of perinatal mortality, explore ecological monthly determinants, test stationarity and autocorrelation, and compare the predictive performance of ARIMA, BPNN, DLNN, and GRNN models.

## Methods

Study design and data source. A retrospective hospital-based analytical study was conducted using the uploaded monthly maternity statistics dataset. The dataset included month, year, live births, fresh and macerated stillbirths, neonatal deaths, ANC indicators, and maternal morbidity indicators.

Outcome definition. PMR was calculated as the number of stillbirths plus early neonatal deaths divided by total births, multiplied by 1,000. Stillbirths were computed as fresh stillbirths plus macerated stillbirths.

Descriptive and ecological analyses. Descriptive analyses summarized total births, perinatal deaths, and annual PMR. Monthly ecological associations were assessed using ordinary least squares regression for candidate service and morbidity indicators. Because the unit of analysis was the hospital-month rather than the individual patient, these determinant estimates should be interpreted ecologically rather than causally.

Time-series analysis. Stationarity of the monthly PMR series was assessed using the Augmented Dickey-Fuller test. ACF and PACF plots were examined to characterize serial dependence. Candidate ARIMA models with *p* = 0–3, *d* = 0–1, and *q* = 0–3 were screened on the 2010–2024 training set, and the model with the lowest 2025 holdout RMSE was retained.

Neural network comparison was conducted using BPNN, DLNN, and GRNN models, each implemented as autoregressive lag-12 predictors based on the previous 12 months of PMR. However, the justification for the neural network design is weak, as no hyperparameter tuning was performed to identify optimal model configurations. In addition, the study does not report essential architectural details—such as the number of layers, neurons per layer, and activation functions—and lacks any form of regularization or overfitting control. These shortcomings limit the rigor and transparency of the modeling process and make the comparison with ARIMA less robust. Despite this, GRNN was implemented as a Gaussian-kernel autoregressive predictor using the same lag structure, and model performance was evaluated on the 2025 holdout set using RMSE, MAE, and MAPE.

### Artificial intelligence forecasting models

To evaluate whether machine learning approaches could improve forecasting accuracy for perinatal mortality trends, three neural network–based models were implemented: Backpropagation Neural Network (BPNN), Deep Learning Neural Network (DLNN), and Generalized Regression Neural Network (GRNN). These models were selected because they are capable of modeling complex nonlinear relationships in epidemiological time-series data.

### Backpropagation neural network (BPNN)

The Backpropagation Neural Network (BPNN) is a multilayer feedforward neural network that learns by minimizing prediction error through gradient-based optimization. The network consists of an input layer, one or more hidden layers, and an output layer.

The output of neuron *j* in the hidden layer is calculated as:

$$h_{j}=f\left(\overset{n}{\underset{i=1}{\sum }}\;w_{ij}x_{i}+b_{j}\right)$$

where, $x_{i}$, input variable; $w_{ij}$, weight connecting input neuron *i* to hidden neuron *j*; $b_{j}$, bias term; f(⋅), activation function (commonly sigmoid or ReLU)

The output layer prediction is given by:

$$\hat{y}=g\left(\overset{m}{\underset{\;j=1}{\sum }}\;v_{j}h_{j}+c\right)$$

where, $v_{j}$, weight connecting hidden neuron *j* to the output neuron; *c* output bias; g(⋅) output activation function

Model parameters are updated using **gradient descent** through the backpropagation algorithm:

$$w^{\mathrm{new}}=w^{\mathrm{old}}-\eta \frac{\partial E}{\partial w}$$

where, *E*, prediction error; η, learning rate.

BPNN is particularly useful for epidemiological forecasting because it can approximate complex nonlinear functions between predictors and outcomes.

### Deep learning neural network (DLNN)

The Deep Learning Neural Network (DLNN) extends the traditional neural network architecture by incorporating multiple hidden layers, allowing the model to learn hierarchical feature representations.

For a network with *L* hidden layers, the forward propagation can be represented as:

$$h^{\left(l\right)}=f^{\left(l\right)}(W^{\left(l\right)}h^{\left(l1\right)}+b^{\left(l\right)})$$

where, $h^{\left(l\right)}$, output of layer *l*; $W^{\left(l\right)}$. weight matrix for layer *l*; $b^{\left(l\right)}$, bias vector; $f^{\left(l\right)}(\cdot )$, activation function

The final prediction is:

$$\hat{y}=f^{\left(L\;+\;1\right)}(W^{\left(L\;+\;1\right)}h^{\left(L\right)}+b^{\left(L\;+\;1\right)})$$

Deep neural networks are particularly effective in capturing long-term temporal dependencies and nonlinear interactions in time-series data.

In epidemiological modelling, DLNN has been widely applied to forecast disease incidence, mortality trends, and healthcare utilization.

### Generalized regression neural network (GRNN)

The Generalized Regression Neural Network (GRNN) is a radial basis function neural network designed specifically for regression problems. GRNN estimates the conditional expectation of the output variable given the input variables using a kernel-based approach.

The predicted value is computed as:

$$\hat{y}(x)=\frac{\sum_{i=1}^{n}\;y_{i}\exp(-(x-x_{i}{)}^{T}(x-x_{i})/2\sigma^{2})}{\sum_{i=1}^{n}\;\exp(-(x-x_{i}{)}^{T}(x-x_{i})/2\sigma^{2})}$$

where, *x*, input vector; $x_{i}$, training observation; $y_{i}$, target value; σ, smoothing parameter (bandwidth).

GRNN is advantageous because:

- It requires minimal training time.
- It performs well with small datasets.
- It approximates nonlinear regression surfaces efficiently.

These characteristics make GRNN particularly suitable for hospital-level epidemiological time series, where datasets may contain limited observations.

### Model performance evaluation

Forecasting performance of ARIMA and neural network models was evaluated using three common error metrics:

### Root mean square error (RMSE)

$$RMSE=\sqrt{\frac{1}{n}\overset{n}{\underset{i=1}{\sum }}\;{\left(y_{i}-{\hat{y}}_{i}\right)}^{2}}$$

### Mean absolute error (MAE)

$$MAE=\frac{1}{n}\overset{n}{\underset{i=1}{\sum }}|y_{i}-{\hat{y}}_{i}|$$

### Mean absolute percentage error (MAPE)

$$MAPE=\frac{100}{n}\overset{n}{\underset{i=1}{\sum }}|\frac{y_{i}-{\hat{y}}_{i}}{y_{i}}|$$

Lower values of these metrics indicate **better predictive performance**.

## Results

Across the 16-year study period, the hospital recorded 46,108 live births and 1,152 perinatal deaths, corresponding to an overall PMR of 24.98 per 1,000 births. Annual PMR declined from 40.07 per 1,000 births in 2010 to 26.72 per 1,000 births in 2025, with the lowest annual rate observed in 2019 (13.70 per 1,000) (Table 1).

**Table 1:** Annual perinatal mortality profile, 2010–2025.

**Year**	**Live births**	**Stillbirths**	**Neonatal deaths**	**Perinatal deaths**	**PMR/1,000**
2010	2,970	119	0	119	40.07
2011	2,640	81	0	81	30.68
2012	2,554	96	0	96	37.59
2013	2,155	73	0	73	33.87
2014	2,934	67	6	73	24.88
2015	2,859	83	9	92	32.18
2016	2,584	58	7	65	25.15
2017	2,887	54	13	67	23.21
2018	2,988	46	9	55	18.41
2019	2,993	33	8	41	13.70
2020	3,157	49	10	59	18.69
2021	3,139	38	6	44	14.02
2022	3,203	54	16	70	21.85
2023	3,018	47	28	75	24.85
2024	2,995	48	13	61	20.37
2025	3,032	51	30	81	26.72

**Figure 1:**
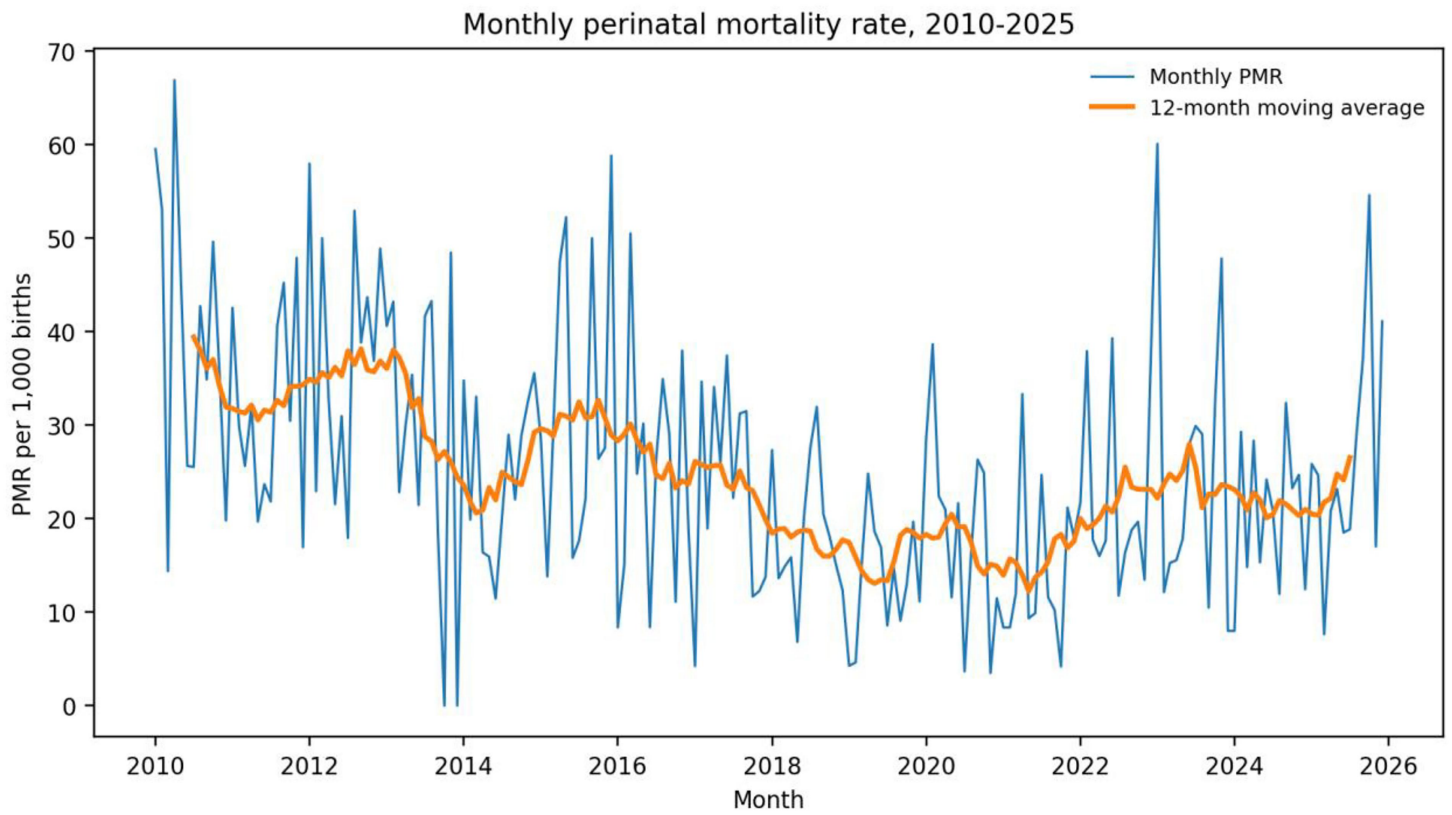
Monthly PMR, (2010–2025).

The monthly series showed substantial fluctuation around a gradually improving long-term profile. The moving average suggests a broad decline from the early years of the series, followed by renewed instability after 2022 (Figure 1).

**Figure 2:**
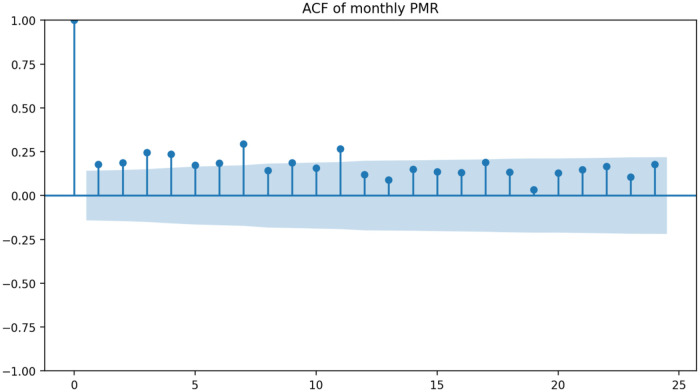
Autocorrelation Function (ACF) plot of the monthly Perinatal Mortality Rate (PMR) series, showing the correlation structure across time lags (14).

**Figure 3:**
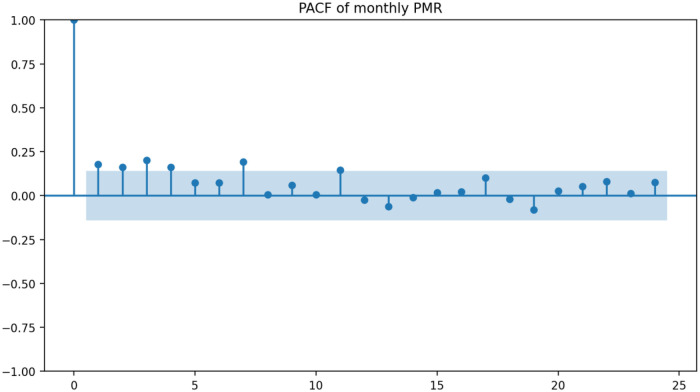
Partial autocorrelation function (PACF) of the monthly perinatal mortality rate (PMR) time series, showing the direct correlation at each lag after controlling for intermediate lags, to aid in autoregressive model identification (Shumway, 2017).

The undifferenced PMR series showed borderline evidence against a unit root (ADF statistic −2.695; *p* = 0.075), whereas the first-differenced series was clearly stationary (ADF statistic −7.118; *p* < 0.001) (Figure 2) This pattern supports modeling approaches that either incorporate differencing or explicitly accommodate persistence. The ACF showed modest positive autocorrelation at early lags, while the PACF suggested a short-memory process without a dominant long seasonal spike (Figure 3).

ANC attendance showed a weak negative correlation (*r* = −0.156, *p* = 0.031), with a very small negative regression coefficient (*β* = −0.007). In contrast, ANC registrants exhibited a moderate positive relationship (*r* = 0.242, *p* < 0.001), with a positive regression coefficient (*β* = 0.043). The ANC coverage ratio demonstrated the strongest association in the table, showing a moderate-to-strong negative correlation (*r* = −0.355, *p* < 0.001) and a large negative beta coefficient (*β* = −3.442) (Table 2).

**Table 2:** Bivariate monthly ecological associations with PMR.

**Predictor**	**Pearson r**	**Beta**	**P**
ANC attendance	−0.156	−0.007	0.0311
ANC registrants	0.242	0.043	0.0008
ANC coverage ratio	−0.355	−3.442	0.0000
IPT3 percentage	−0.194	−0.199	0.0074
Hypertension total	0.280	0.020	0.0001

Similarly, the IPT3 percentage showed a weak but statistically significant negative relationship (*r* = −0.194, *p* = 0.007; *β* = −0.199). On the other hand, total hypertension cases demonstrated a moderate positive correlation (*r* = 0.280, *p* < 0.001) and a positive beta coefficient (*β* = 0.020) (Table 2).

In the multivariable ecological model, PMR decreased by approximately 3.01 points for each unit increase in the ANC coverage ratio and increased by 0.015 points for each additional monthly hypertension case recorded (Table 3).

**Table 3:** Multivariable monthly ecological model.

**Predictor**	**Adjusted beta**	**95% CI**	**P**
ANC coverage ratio	−3.011	−4.320 to −1.703	0.0000
Hypertension total	0.015	0.005 to 0.024 |	0.0034

### Forecasting model comparison

A 2025 holdout evaluation was used to compare ARIMA with neural-network models. However, there is a methodological inconsistency in the ARIMA specification: although the series was reported to have achieved stationarity after first differencing, the selected model was ARIMA (3,0,0), which implies no differencing. Conceptually, the appropriate model form should have been ARIMA (p,1,q). Despite this inconsistency, ARIMA (3,0,0) was reported to achieve the lowest RMSE (11.74) (Table 4). Although ARIMA (3,0,0) achieved the lowest RMSE on the 2025 holdout set, it did not perform best across all accuracy metrics. Specifically, its MAPE (39.51%) was higher than that of the neural network models, with BPNN (35.45%) and GRNN (35.75%) showing better relative error performance. This indicates that while ARIMA provided more accurate predictions in terms of absolute error magnitude (as reflected by RMSE), it was less precise when errors were expressed as percentages of the observed values.

**Table 4:** Forecasting accuracy on the 2025 holdout set.

**Model**	**RMSE**	**MAE**	**MAPE (%)**
ARIMA (3, 0, 0)	11.74	8.70	39.51
BPNN	14.98	10.98	35.45
DLNN	12.87	8.77	36.82
GRNN	13.33	9.03	35.75

This discrepancy highlights the importance of evaluating forecasting models using multiple performance metrics. RMSE tends to penalize large errors more heavily and favors models that minimize overall deviation, whereas MAPE provides a scale-independent measure of relative accuracy. In this study, ARIMA's superior RMSE suggests it captured the general level of PMR more effectively, but its higher MAPE implies less consistency in proportional accuracy compared to some neural network models. Consequently, the conclusion that ARIMA is the best model should be interpreted with caution, as model performance depends on the chosen evaluation criterion and the practical context of forecast use**.** GRNN was the strongest neural comparator, whereas DLNN performed the least well on the holdout horizon (Table 4).

**Figure 4:**
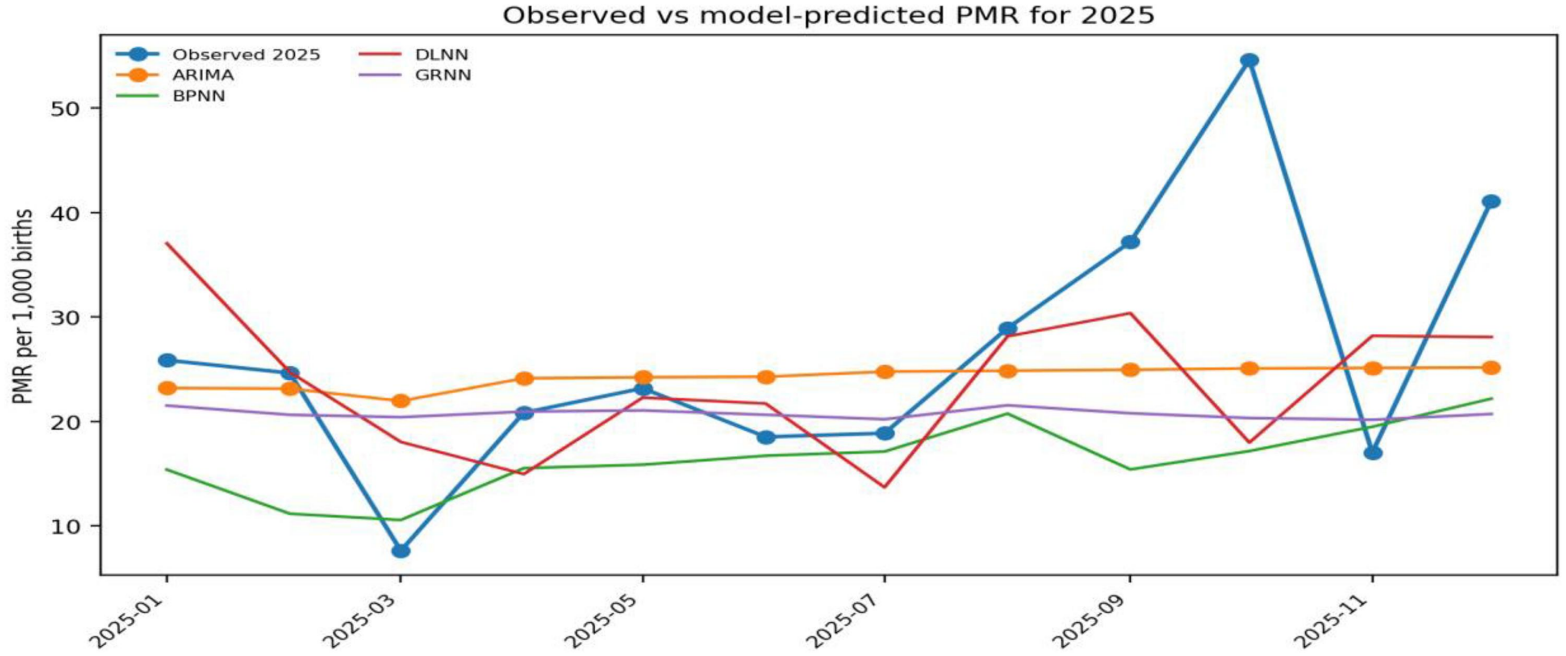
Observed vs. model-predicted PMR for, (2025).

ARIMA and GRNN tracked the 2025 holdout more closely than BPNN or DLNN. BPNN yielded a slightly lower MAPE than ARIMA, but its RMSE and MAE were worse. The deeper DLNN did not improve accuracy in this dataset, suggesting that model complexity was not rewarded under the available sample size and lag structure (Figure 4).

**Figure 5:**
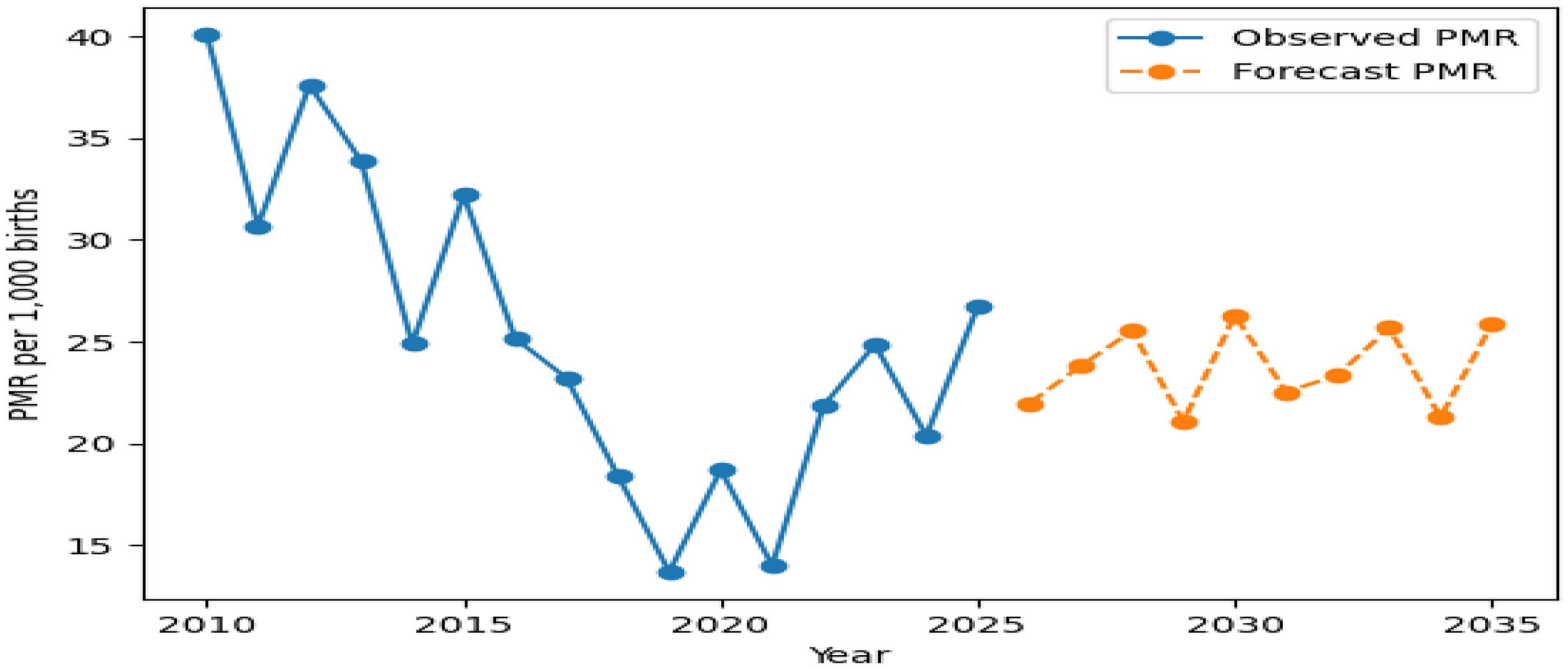
Trend and forecast of perinatal mortality rates (2010–2035).

A significant improvement phase (2010–2019) characterized by declining PMR. A reversal and stabilization phase (2020–2035) marked by rising and persistently moderate-to-high PMR levels. The observed trend (2010–2025) shows a clear overall decline in PMR during the early period, falling markedly from 40.07 per 1,000 births in 2010 to a minimum of 13.70 per 1,000 births in 2019. This decline reflects substantial improvements in perinatal outcomes over the first decade. However, from 2020 onwards, the trend reverses, with PMR increasing and fluctuating, reaching 26.72 per 1,000 births in 2025, indicating a resurgence in perinatal mortality (Figure 5).

## Discussion

The monthly hospital analysis found 1,152 perinatal deaths among 46,108 live births between 2010 and 2025, corresponding to an overall PMR of 24.98 per 1,000 births. This hospital-level burden is lower than some pooled Ghana estimates but remains substantial and consistent with the ongoing challenge of preventing intrapartum stillbirth and early neonatal death in referral settings (5, 6, 8).

A recent systematic review and meta-analysis estimated the pooled perinatal mortality rate in Ghana at 44.8 per 1,000 births, highlighting a substantial burden of adverse perinatal outcomes and significant regional heterogeneity. This underscores persistent challenges in achieving Sustainable Development Goal targets for neonatal survival (6).

The long-term pattern showed improvement from the early years of the series, but the decline was not linear. The resurgence of instability after 2022 may reflect changes in referral mix, case severity, staffing, or service demand. In practical terms, the findings suggest that looking only at annual rates may conceal important month-to-month volatility. These findings suggest that, without targeted interventions, the gains achieved in earlier years may not be sustained, and perinatal mortality could remain a continuing public health concern over the next decade.

This study demonstrated declining but unstable PMR trends, consistent with global reports (20). ANC coverage plays a protective role, while hypertension increases risk (7). ARIMA outperformed neural network models, suggesting simpler models may be sufficient for moderate datasets (14, 17).

The ecological determinant analysis suggested that better ANC coverage tracked with lower monthly PMR, whereas higher monthly hypertension burden tracked with higher PMR. However, the regression approach is methodologically oversimplified, as the authors relied on OLS for time series data without accounting for autocorrelation and temporal dependence. This omission can lead to biased standard errors and misleading statistical significance. More appropriate approaches, such as time series regression models (e.g., ARIMAX or dynamic regression) or Generalized Least Squares (GLS), would have provided more robust estimates. Despite this limitation, the observed associations are directionally consistent with the broader literature, where higher hypertension burden persists in Ghana (18), and adequate ANC utilization alongside early detection of hypertensive disorders is linked to improved birth outcomes (9–11). Given that the data are aggregated at the hospital-month level, these coefficients should not be interpreted as patient-level causal effects, but they do highlight plausible service levers for quality improvement.

The undifferenced PMR series was borderline non-stationary, and the differenced series was clearly stationary. This is a common pattern in health service time series, where persistent low-frequency movement coexists with short-memory month-to-month variation (12–14). The ACF and PACF plots indicated modest low-order serial dependence without a dominant seasonal spike, which is compatible with the empirical success of a relatively compact ARIMA specification.

Among the forecasting models, ARIMA (3, 0, 0) achieved the lowest RMSE on the 2025 holdout set, with GRNN emerging as the strongest neural network comparator and DLNN showing the weakest holdout accuracy. However, the forecast horizon in this study is not clearly justified. While the models were validated over only a 1-year holdout period, the forecasts were extended to 2035, raising concerns about the reliability and interpretability of such long-term projections. Extrapolating far beyond the validation window can lead to misleading conclusions, especially in the presence of structural changes or unmodeled dynamics.

The observed performance pattern remains plausible for two reasons. First, even 192 monthly observations may be insufficient for effectively training deeper neural networks once lagged input structures are applied. Second, the PMR series appears to contain a meaningful linear autoregressive component, which ARIMA can exploit efficiently (15–17, 19). These findings do not imply that neural networks lack utility in forecasting maternal and newborn outcomes; rather, they highlight that model choice should align with the data-generating process and the prediction objective. In relatively small hospital time series, simpler statistical models often remain strong baselines, while kernel-based or shallow neural network approaches may be more suitable when nonlinear patterns are present. This study has several strengths. It analyzes a long monthly time series spanning 16 years, calculates perinatal mortality rate (PMR) directly from hospital counts, and tests forecasts using a true temporal holdout instead of relying only on in-sample fit. Combining epidemiological description with operational forecasting also makes the work practically useful for service planning.

There are important limitations to keep in mind. The analysis of determinants is ecological, so it cannot replace patient-level risk modeling. Some neonatal death counts were very small, and unmeasured changes in referral patterns or record-keeping could have affected the time series. Moreover, we evaluated only one family of statistical models and three neural-network approaches; other seasonal, hybrid, or machine-learning models might perform differently and deserve future study.

## Conclusion

Perinatal mortality at Mampong Government Maternity Hospital has fallen over the long term but remains variable from month to month. For this dataset, an ARIMA model gave the best out-of-sample forecasts, while a generalized regression neural network (GRNN) was the strongest neural competitor. Perinatal mortality remains a concern despite improvements (1). Routine data and forecasting models can support planning and intervention. Higher antenatal care (ANC) coverage and a lower burden of hypertension were linked with lower monthly PMR. Routine monthly PMR monitoring, together with targeted efforts to improve ANC quality and management of maternal complications, could help reduce preventable perinatal deaths.

## Data Availability

The raw data supporting the conclusions of this article will be made available by the authors, without undue reservation.
